# Phenylpropanoid-enriched broccoli seedling extract can reduce inflammatory markers and pain behavior

**DOI:** 10.1186/s12967-023-04777-1

**Published:** 2023-12-19

**Authors:** Aleksandra A. Gurgul, Yahya Najjar, Ana Chee, Howard An, Chun-Tao Che, Thomas J. Park, Katherine M. Warpeha

**Affiliations:** 1 Department of Pharmaceutical Sciences, College of Pharmacy, University of Illinois Chicago, Chicago, IL USA; 2 Department of Biological Sciences, University of Illinois Chicago, 900 S Ashland Ave, M/C 567, Chicago, IL 60607 USA; 3 Department of Orthopedic Surgery, Rush University Medical Center, Chicago, IL USA

**Keywords:** Anti-inflammatory, Phenylpropanoid, Polyphenol, Abiotic stress, Extract, Seedling, Broccoli, Non-opiate, Inflammatory pain, Natural products

## Abstract

**Background:**

Pain is a worldwide problem requiring an effective, affordable, non-addictive therapy. Using the edible plant broccoli, a growth protocol was developed to induce a concentrated combinatorial of potential anti-inflammatories in seedlings.

**Methods:**

A growth method was utilized to produce a phenylpropanoid-rich broccoli sprout extract, referred to as Original Extract (OE). OE was concentrated and then resuspended for study of the effects on inflammation events. A rabbit disc model of inflammation and degeneration, and, a mouse model of pain behavior were used for in vivo and in vitro tests. To address aspects of mammalian metabolic processing, the OE was treated with the S9 liver microsome fraction derived from mouse, for use in a mouse in vivo study. Analytical chemistry was performed to identify major chemical species. Continuous variables were analyzed with a number of methods including ANOVA, and two-tailed *t* tests, as appropriate.

**Results:**

In a rabbit spine (disc) injury model, inflammatory markers were reduced, and levels of regenerative markers were increased as a result of OE treatment, both in vivo and in vitro. In a mouse pain behavioral model, after treatment with S9 liver microsome fraction, the resultant extract significantly reduced early and late pain behavior in response to a pain stimulus. The OE itself reduced pain behavior in the mouse pain model, but did not achieve the level of significance observed for S9-treated extract. Analytical chemistry undertaken on the extract constituents revealed identities of the chemical species in OE, and how S9 liver microsome fraction treatment altered species identities and proportions.

**Conclusions:**

In vitro and in vivo results indicate that the OE, and S9-treated OE broccoli extracts are worthwhile materials to develop a non-opiate inflammation and pain-reducing treatment.

**Supplementary Information:**

The online version contains supplementary material available at 10.1186/s12967-023-04777-1.

## Background

Pain affects over 100 million people a year in the USA and is a human problem worldwide. Current non-opiate medications, such as acetaminophen or non-steroidal anti-inflammatory drugs (NSAIDs), are inadequate for pain management, while opiates have specific drawbacks including, lack of efficacy, toxic side effects with long-term use, tolerance, and addiction [1–5]. Narcotic-related deaths still occur at high rates, despite the reduction of prescriptions written [6]. Narcotic-related overdoses and deaths continue to worsen in the U.S. [7]. A key feature of pain is a detrimental inflammatory phenotype, presenting higher levels of pro-inflammatory and lower levels of anti-inflammatory cytokines, often involving neuronal dysfunction [8–14]. The inflammatory basis of pain is supported by data from human subjects [15–18] and transgenic animal models [19]. We propose a novel strategy to target pathways that can lead to inflammation and subsequent pain, by taking advantage of inducible plant defensive responses.

Plants are sessile organisms that have developed intricate and reliable phytochemical defense mechanisms to environmental stimuli. Induced and activated by environmental signals (e.g. UV-light, chilling), phytochemicals (typically phenylpropanoids i.e. derived from phenylalanine) are involved in several functions that include scavenging of free radicals and reducing resultant stress-derived toxins [20, 21]. A growing body of evidence indicates that plant phenylpropanoids also reduce or prevent inflammation in mammals in vitro and in vivo [21]. Phenylpropanoids can beneficially alter cellular processes in inflammation and pain-associated diseases [22]. Some secondary metabolites possess anti-inflammatory and neuroprotective properties, but polyphenols, in particular, synergistically induce anti-stress functions [20, 21, 23, 24]. However, in commonly eaten plants, chemicals that would contribute to combating pain conditions are not present in sufficient quantities [25], and/or some molecules appear to require association with other chemicals to be effective, where single isolates fail to address inflammation and pain [20, 23, 26–32]

In Arabidopsis, a genetic model for plant metabolism, mutants were identified that produced phenylpropanoids that reduced plant cellular stress [33–35]. Growth conditions have been refined for phenolic induction [35, 36], then tested in stressed primary cultures derived from a mouse APOE gene replacement model of low, average, and high risk of developing Alzheimer’s Disease (AD) [15]. Data indicated that extracts reduced negative aspects of the inflammatory response, especially in the difficult-to-treat carriers of APOE4 alleles [15].

Herein, we have developed an inducible, stable anti-inflammatory plant chemical profile derived from an abiotically stimulated compact non-genetically modified broccoli heirloom cultivar. Growth methods and experimental extractions led to an extract of mainly phenylpropanoids that were stable post-harvest. Extracted chemicals were identified by analytical chemistry. Experiments were conducted in both a rabbit spine model and a mouse behavior model of pain indicating that anti-inflammatory activity was in evidence. The extract was processed to increase bioavailability, and indicated effectiveness for future non-narcotic, potential pain alleviation therapies.

## Methods

### Chemicals

All chemicals used for plant growth and preparation of extract were obtained from Sigma-Aldrich (St. Louis, MO, USA), and Thermo Fisher Scientific (Waltham, MA USA).

### Plant material

Planting and preparation of plant material were performed as previously described [15], but are detailed here for clarity. Broccoli seeds (*Brassica oleracea* var. Italica, cv obtained from Eden brothers (Arden, NC) and Johnny’s Selected Seeds (Winslow, ME)) were sown (500 seeds/tray) and grown in complete darkness on 0.5X Murashige and Skoog media 0.8% agarose phytatrays, then sealed in black Plexiglas boxes as described [37]. Cold-vernalized phytatrays were then moved to continuous darkness at 20 °C for 5 d [37]. Plant growth and treatment procedures were performed as previously described [15], with the following modifications. Broccoli seedlings were treated with sublethal UV treatments of 254 nm, and 315 nm [34], each at a fluence (dose) of 10^4^ μmol m^−2^, followed by cold (1 h at 4 °C) and blue light (10^4^ μmol m^−2^), with 1 min between treatments on d 5, then phytatrays returned to darkness for 12 h. In the first instance the primary leaves were harvested live in to 90% ethanol for maceration, then allowed to sit 1 h at 4 °C. Debris was pelleted and supernatant read by spectrophotometer (SmartSpec™ Bio-Rad, Hercules, CA USA) where heirloom cultivars were checked for content of sinapates and flavonoids based on conditions for extraction [38]. Calabrese, an heirloom broccoli became the focus for this study. Other heirloom cultivars utilized in the fast screen are shown in Additional file 1: Table S1. Calabrese seeds were sown and grown and treated as described above, then 12 h post treatments, the upper hypocotyl and leaf material were harvested directly into liquid nitrogen, ground to powder then stored at − 80 °C. Samples were maintained at − 80 °C until extracted (referred to as OE) and then treated to make S9-treated OE (described below) to mimic liver processing.

### Plant material extraction

Extraction was performed as previously described with some modifications [37]. The aerial portions of Calabrese ground in liquid nitrogen were weighed and extracted twice for two h with 95% aqueous ethanol by sonication (20 mL of solvent per gram of plant material). After filtration, the filtrate was evaporated to remove the solvent using a rotary evaporator under a reduced vacuum, then freeze-dried. Freeze dried material (150 mg/mL) was resuspended in DMSO 1:2 for storage, and later DMSO samples were further diluted (referred to as OE) as described in PBS for experiments, with 1:200 dilution used for mouse behavior studies being 375 μg/mL.

### Rabbit disc cell isolation, culture and treatment

Rabbit disc tissues were isolated from New Zealand white rabbits spines, obtained from the animal tissue program at Rush where investigators donate unused tissues from one study to others to help conserve the number of animals used in research. Cells were isolated by sequential enzymatic digestion, seeded in tissue culture plates and grown in monolayers in complete media [DMEM/F12 (Mediatech, Manassas, VA, USA) supplemented with 50 µg/mL gentamicin (Gibco, Thermo Fisher Scientific), 25 μg/mL ascorbic acid (Sigma-Aldrich, St. Louis, MO, USA) and 20% fetal calf serum (Omega Scientific, Tarzana, CA, USA)] in a 5% CO_2_ incubator at 37 °C. After cells reached 80% confluency, they were cultured for 24 h in starvation media [DMEM/F12 supplemented with 1% insulin, human transferrin, and selenous acid (ITS, Corning Life Sciences, Tewksbury, MA, USA), L-glutamine, gentamicin and ascorbic acid], and then treated with lipopolysaccharide (LPS; 100 ng/mL; Sigma) in the presence or absence of different concentrations of broccoli OE for 16 h. Control treatment consisted of starvation media alone and was included in all experiments. Conditioned media were collected for protein analysis and cell pellets for RNA analysis.

### Conditioned media analysis of rabbit Interleukin-8 (IL-8)

Conditioned media were collected and stored at − 80 °C until analysis. Rabbit IL-8 levels were measured using enzyme-linked immunosorbent assay kit for rabbit IL-8 (Raybiotech, Norcross, GA, USA) following the manufacturer’s instructions.

### Surgical methods for rabbit disc degeneration and treatment

New Zealand white rabbits (Charles River Laboratories, Wilmington, MA, USA) weighing about 2.8–3.0 kg were used in this study (n = 8 total). The rabbits were cared for and maintained in accordance with National Institute of Health guidelines. All studies were approved by Rush University Medical Center’s Institutional Animal Care and Use Committee (#14-064), and have been described previously [38–45]. Under general anesthesia and using aseptic conditions, a left abdominal incision was made and the ventral surface of four consecutive lumbar IVDs (L3/4, L4/5, and L5/6) was exposed. Using an 18-gauge needle with a stopper device that allows the needle to go to a depth of 5 mm, the annulus fibrosus was punctured in the ventral aspect into the nucleus pulposus (NP) at all 3 levels. Suction was applied for the removal of NP tissues. Rabbits were randomized and discs were treated during the same operation of puncture. Eight microliters (8 μL) of OE (n = 4) or saline (0.9% Sodium Chloride, Baxter Healthcare Corporation, Deerfield, IL USA; n = 4) were injected into the center of the injured discs with a 26-gauge needle. The surgical wound was repaired in layers. Meloxicam (1.5 mg) was also given orally (one day [d] before surgery and 2–3 d after the operation). An analgesic (buprenorphine HCl 0.01–0.03 mg/kg) was given up to twice daily for 2–3 d, when needed, in consultation with the veterinary staff. After recovery from anesthesia, we returned the rabbits to their cages where they mobilized ad lib. At 3 weeks post-surgery and treatment, animals underwent euthanasia, and discs were isolated for RNA analysis.

### Isolation of total RNA and measurement of mRNA levels with real-time polymerase chain reaction (real-time PCR)

Total RNA was processed from cell pellets and intervertebral disc tissues. RNA isolation and real-time PCR analysis was performed as described in previous studies [41]. Taqman gene expression assays were used for analysis or normalization of the following genes: rabbit collagen type I (COL1), collagen type II (COL2), IL-8, hypoxanthine phosphoribosyltransferase 1 (HPRT1) and 18 S rRNA (Oc03396113_m1, Oc03396134_m1, Oc03397860_m1, Oc03399461_m1, Hs99999901_s1; all from Applied Biosystems, Foster City, CA). PrimeTime® Mini qPCR Assays were designed for rabbit nitric oxide synthase 2 (NOS2), CCL2, and CCL5 (Integrated DNA Technologies, Coralville, IA USA) as previously described [42]. DataAssist™ Software was used to calculate the relative gene expression using the comparative CT (ΔΔCT) method.

### Treatment of OE using S9 liver microsome fraction

The S9 fraction (S9) is a sub-cellular fraction of liver microsomes that contains both phase 1 and 2 metabolizing enzymes [46]. To mimic liver processing and to improve bioavailability [46], we treated the OE with mouse liver S9 fractions (Gibco, Thermo Fisher Scientific) according to the manufacturer’s instructions with some modifications, using the protocol at four times the recipe (volumes). Solubilized OE at 1:2 dilution (75 mg/mL) was added as the ‘test article’ volume × 2 so the total of OE was 4 μL in the assay (total single assay volume is 200 μL). After the supernatant was removed, we spun one additional time and saved the supernatant which was 100 μL, approximately a 50-fold dilution of the OE. This treated extract was labeled the S9-treated OE, and was stored in − 20 °C. When used in a mouse behavior study, the S9-treated OE was diluted in PBS to be equivalent to 1:200 dilution (375 μg/mL) of extract, with ip of 20 μL. No organic solvent was used in the preparation of S9-treated OE. The S9-treated OE was utilized in experiments with live mice in vivo at RT.

### Mouse activity and pain studies

*Activity.* Six-month-old C57BL/6 J adult male (n = 4) and female (n = 4) mice (Charles River Laboratories, Wilmington, MA USA) were used for general activity monitoring, and pain behavior tests as a result of formalin. Animals were kept in a temperature-controlled environment of 22 °C with a 12 h light/dark cycle [47]. All procedures were conducted according to the animal protocols approved by the University of Illinois at Chicago Institutional Animal Care and Use Committee. To control for the possibility that the extract might reduce activity in general and not specifically pain behaviors, we used the open-field paradigm as a measure of activity [48]. We placed mice one at a time into an opaque plexiglass arena and allowed them to freely explore. The arena floor was demarcated into 36 (6 × 6) equal square regions of 5 × 5 inches, or 12.5 cm each. We recorded the number of times a mouse completely crossed from one region to another over a 10 min period. On d 1, half of the mice were given OE (20 μL) via ip injection. The other half of the mice were given an equal volume (20 μL) of vehicle control. Testing commenced 30 min later. On d 2, the mice that had been given OE received vehicle, and the mice that had been given vehicle received OE. We used a paired t-test to compare the number of regional crossings for extract treatment versus vehicle treatment. There was no difference in activity for VC and OE. Hence, data in response to pain stimulus is not a result of activity changes.

#### Pain tests

All mice were randomized, then received either intraperitoneal (ip) 20µL (1:200; 375 µg/mL) extract or vehicle control (VC) (1:200 DMSO in sterile PBS) 30 min prior to subcutaneous injection of 20µL (2%) formalin to the hind paw using a 30-gauge insulin syringe. The formalin test was performed as previously described with modifications [47, 49]. Upon injection, the timer started for behavioral observations. Animals were placed into a standard empty mouse cage and observed for 70 min. Licking of the formalin-injected foot was operationally defined as pain behavior. The total time was divided into 0–5 min (Phase I) defined as early phase, and 40–55 min (Phase II) defined as late phase. The test was performed and scored by observers blinded to experimental conditions. Pain behavior tests were performed on two different days with a 4-d gap between VC or extracts. Animals were randomized into treatment (OE or S9-treated OE) or control groups and switched for testing after the 4-d gap.

### Statistical analyses

All statistical analyses were carried out using GraphPad Prism v. 9 (GraphPad Software, San Diego, CA, USA), with suggested tests from GraphPad Prism after determination that data followed a normal distribution. For all tests, differences were considered significant when the P value was less than 0.05 (P < 0.05). *Rabbit experiments*. Gene expression and protein analysis were analyzed between treatment groups with One way ANOVA, with multiple comparisons. In vivo rabbit disc gene expression was analyzed with multiple unpaired t-tests. *In vivo mouse data*. All data in this paper are represented as mean ± SEM. Unpaired t-test with Welch’s correction was performed for the groups in the mouse behavior tests (early and late pain behavior), where vehicle control was compared to treatment (OE or S9-treated OE) for early and late responses as shown on the figures. *P < 0.05, **P < 0.01, ***P < 0.001.

### HPLC and mass spectrometry (MS)

Pre- and post-treatment extracts were analyzed using HPLC–UV-MS/MS to identify the chemical constituents. The LC–MS method was developed based on the previously described analysis [15]. Sample preparation involved removal of solvents from pre- and post-treatment extracts (DMSO and PBS, respectively) by water wash on a C18 cartridge, followed by sample elution with methanol. LC–MS samples were prepared in LC–MS-grade methanol and filtered through 0.2 µm membrane filters. A Shimadzu UFLC system coupled with an LCMS-2020 detector, and an Agilent EC-C18 column (3.0 × 150 mm, 2.7 µm) protected with a pre-column (3.0 × 5 mm), were used for the HPLC–UV-MS analyses. 0.1% formic acid in water (A) and 0.1% formic acid in acetonitrile (B) were used as mobile phase and run under the gradient program as follows: 5% B (0–1 min), 5–15% B (1–2 min), 15–30% B (2–29 min), 30–100% B (29–30 min), 100% B (30–35 min). Flow rate was maintained at 0.4 mL/min and column oven temperature was set at 27 °C. The LC–MS/MS analyses were performed on a Bruker Impact II quadrupole/time-of-flight (Q/TOF) mass spectrometer coupled with a Shimadzu UHPLC system under similar chromatographic conditions. The bar charts were created using Origin 2020, Version 9.7 (OriginLab Corporation, Northampton, MA, USA) software.

## Results

### Production of a novel and stable broccoli extract producing specific phenylpropanoids

Sprouts have many concentrated nutrients naturally [50], and broccoli, in particular, has been studied in the case of sulforaphane [51, 52]. After testing various abiotic stimuli, we developed a 5-day-sprout protocol that deliver increased phenylpropanoids, determined by experimental extraction methods. The optimal extraction was an ethanol-based method [15], where the broccoli sprouts presented a high ratio of flavonoids and sinapates (Additional file 1: Figure S1). This original extract, abbreviated OE, was resuspended in DMSO, then subsequently diluted in sterile PBS as described in Methods, compared to the vehicle control (VC). Both OE and a VC were tested in rabbit and mouse experimental models of inflammation, described herein.

### Broccoli extract OE inhibits inflammation in rabbit disc cells, and may induce disc regenerative markers in a rabbit model

**Fig. 1:**
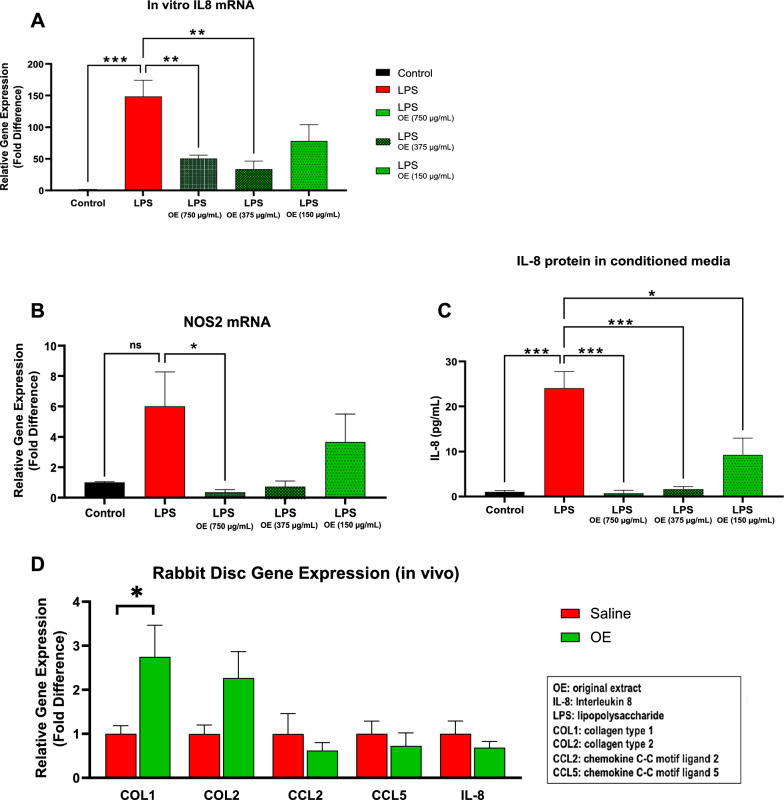
OE reduces inflammation in rabbit disc cells in vitro, increases collagen gene expression in vivo. **A**–**C** Rabbit disc cells were pretreated with different concentrations of OE and then stimulated with lipopolysaccharide (LPS) for 16 h. Gene expression of Interleukin-8 (IL-8) and nitric oxide synthase 2 (NOS2) was analyzed. Statistically significant differences between OE-treated and VC (saline) from LPS (P < 0.05) are indicated (*). Conditioned media was collected to quantify interleukin-8 (IL-8) protein. (Lower panel D) Rabbits (n = 8) underwent surgical disc injury and intradiscal treatment with OE or VC. After 3 weeks, intervertebral disc tissues were isolated for RNA analysis of phenotypic disc and inflammatory genes: collagen type I (COL1), collagen type II (COL2), chemokine C–C motif ligand (CCL)2, CCL5 and IL-8 (**D**). Cell culture experiment data were analyzed with One way ANOVA with multiple comparisons and included in the significant difference (*) on graphs. In vivo, rabbit disc gene expression was analyzed with multiple unpaired t-tests. Error bars represent SEM

Well-established in vitro studies with disc cells have shown that exposure to lipopolysaccharide (LPS) or inflammatory cytokines can initiate an inflammatory response that leads to the upregulation of catabolic genes associated with disc degeneration [53]. Previous studies have also shown that treatment with growth factors can decrease catabolism in disc cells [54]. To determine any anti-inflammatory effects of OE on disc cells, rabbit disc cells were pre-treated with different concentrations of OE and then exposed to LPS. OE had a dose-dependent effect on reducing gene expression of inflammatory markers interleukin-8 (IL-8) and nitric oxide synthase 2 (NOS2) (Fig. 1A, B), and in decreasing the release of IL-8 protein into the conditioned media (Fig. 1C).

A rabbit disc injury model may promote understanding biological mechanisms of disc degeneration and test therapeutics that would help regenerate the disc such as growth factors, cell therapies, and anti-inflammatory molecules. Rabbits (n = 8) underwent disc-injury surgery to induce disc degeneration and inflammation and intradiscal treatment with either saline (control) or OE. After 3 weeks, animals were sacrificed, and disc tissues were isolated for gene expression analysis for disc phenotypic and inflammatory markers. OE-treated injured discs showed higher expression of collagen type 1 (COL1) and collagen type 2 (COL2), indicating disc regeneration. Discs treated with OE indicated decreases in inflammatory gene expression chemokine C–C motif ligand (CCL)2, CCL5, and IL-8, but did not achieve significance (Fig. 1D).

### Mouse in vivo pain model indicates that OE and S9-treated OE extract (S9) are effective at reducing pain behavior

**Fig. 2:**
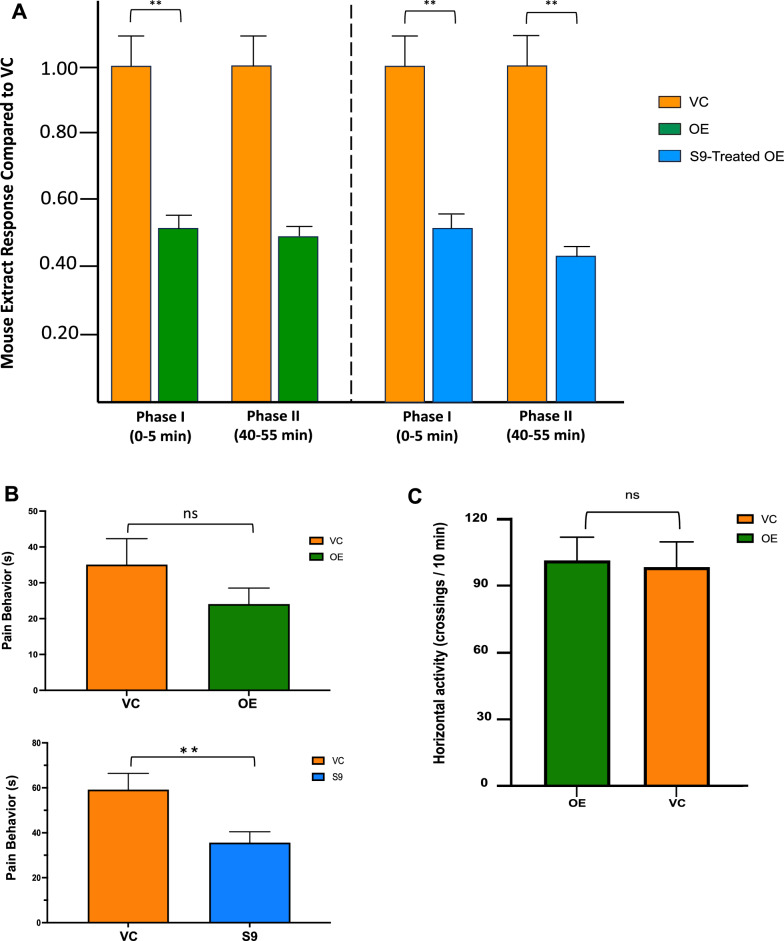
Phenylpropanoid-enriched broccoli extracts reduce pain behavior in mice. **A** OE was administered as an ip (20 μL, 375 μg/mL) treatment 30 min before formalin injection to the paw. At time 0 min, mice were injected in the rear left dorsal side of the paw with formalin as described in the Methods, and timing commenced for pain behavior (licking the injured paw); early (0–5 min) and late (40–55 min) are shown. For OE-treated mice, significantly reduced pain behavior in mice was apparent only during the early Phase I [0–5 min; P = 0.0032 (n = 8)], and was not significant in the later phase [Phase II; 40–55 min (shown on Left)]. VC is set to 1.00 for comparison of OE, and for the S9-treated OE. S9-treated OE (20 μL, 375 μg/mL) was significant for both early and late behavior as shown on the Fig.A (phase I P = 0.0018; phase II P = 0.0062). **B.** Pooled male and female mice pain behavior over the 0–70 min period indicated that reduction in pain (licking of injured foot) was significant with S9-treated OE but not OE. An unpaired t-test with Welch’s correction resulted in a P-value of .0012 for S9 and .2096 for OE (n = 8). **C** Mice were injected at T = − 30 min with OE. At zero, mice were allowed to explore in their activity maze as described in Methods. OE showed no significant changes in general mice activity, eliminating possible confounding variables in the assessments of pain reduction

We utilized an accepted pain model for mammals’ reflexive and non-reflexive inflammation, utilizing formalin as the pain stimulus [13, 47, 49, 55]. To start the experiment, 30 min prior to pain stimulus, a 20 μL ip injection of OE or S9-treated OE (375 µg/mL) or vehicle control (VC) was administered to male and female mice. At time zero, the left dorsal hind paw of each mouse was given a subcutaneous injection of formalin (20 μL of 5 mg/mL in 0.1 M PBS). At injection, a dedicated timer was started at time 0 s to record the recognized pain behavior of paw-licking at the site of injection for 70 min, counted in brackets of 5 min (0–5, 5–10 min, etc.), to reveal early and late behavior [47, 49], shown in Fig. 2. OE-treated mice were evaluated for this pain test (4 female, 4 male; total n = 8). Pooling the male and female mouse data obtained for OE revealed the early response (Phase I, 0–5 min) was significant (P = 0.0032), but not the later response (Phase II, 40–55 min; P = 0.2182) where VC response was set to 1.0, shown in Fig. 2A. S9-treated OE was evaluated for the same in vivo pain test. Male and female responses were pooled for comparison of S9-treated OE to VC pain response data for early (Phase I, 0–5 min; P = 0.0018) and late (Phase II, 40–55 min; P = 0.0062) behavior with VC set to 1.0 (Fig. 2A). Sex differences are possible in pain responses, but when male and female data were separated and compared, no sex differences were observed for either OE early (Phase I, 0-5 min; VC P = .2521, OE P = .1509) and late (Phase II, 40-55 min; VC P = .1815, OE P = .8056), or S9-treated OE for early (Phase I, 0-5 min; VC P = .7941, S9 P = .9848) and late (Phase II, 40-55 min; VC P = .1921, S9 P = .9828) pain behavior (Fig. S2). To better understand the overall responses of pooled male and female mice over the 0–70 min testing period, OE and S9-treated OE responses were compared to VC, where S9-treated OE was significant (P = 0.0012), but OE was not (P = 0.2096) shown in Fig. 2B. With a different set of mice (4 female, 4 male) of same age, the OE was tested to ensure there were no alterations of basic activity, where differences between VC and OE were ns (Fig. 2C).

### Chemical constituents in extracts indicate good candidates for anti-inflammatories and reveal processing information

**Fig. 3:**
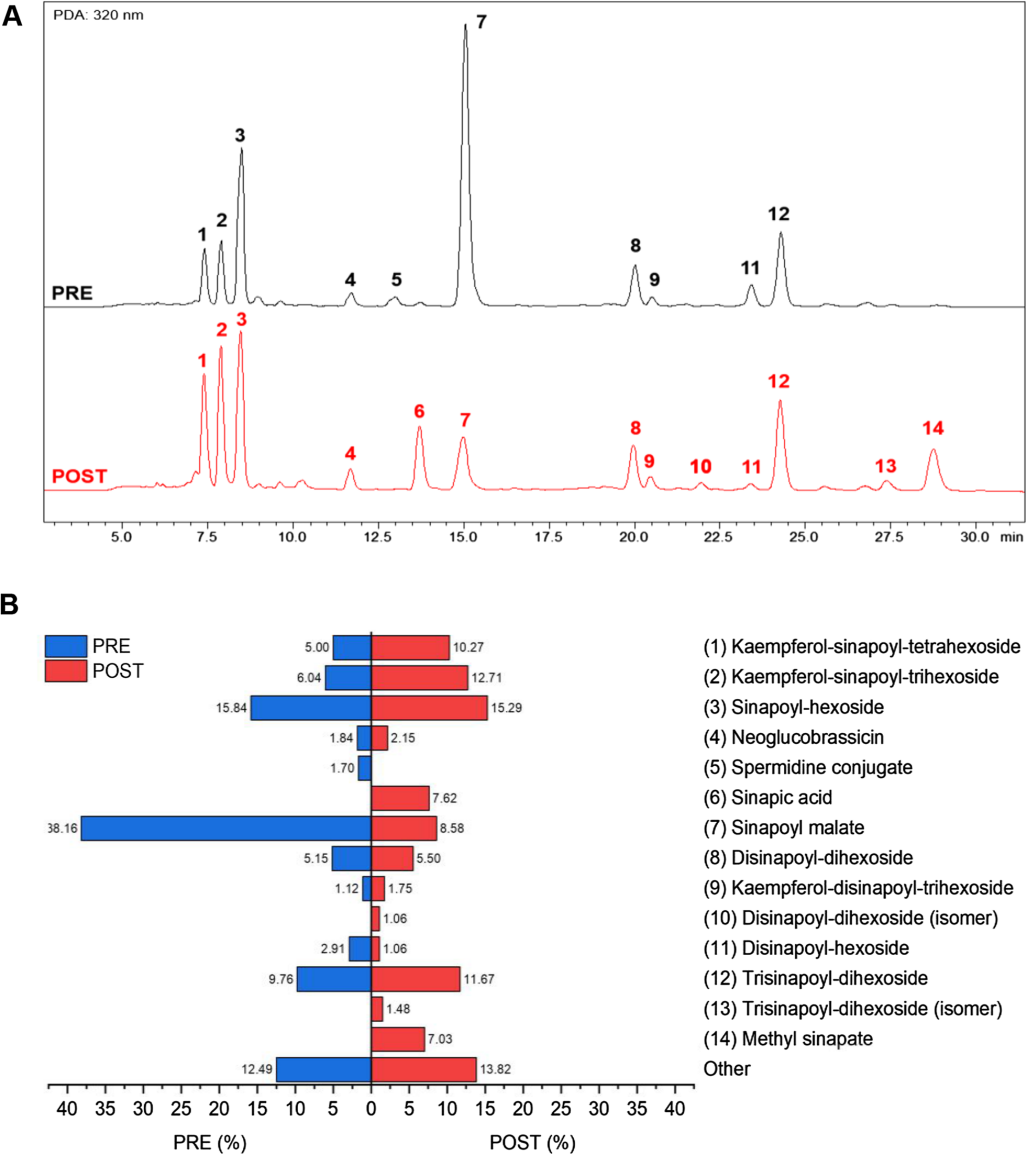
Identification of chemical constituents of broccoli extract OE, pre- and post-treatment with S9 fraction. **A** HPLC chromatograms recorded under UV at 320 nm show component peaks (numbered 1–14) in the broccoli extracts pre- (undigested OE) and post-treatment (S9). Peaks: (1) Kaempferol-sinapoyl-tetrahexoside; (2) Kaempferol-sinapoyl-trihexoside; (3) Sinapoyl-hexoside; (4) Neoglucobrassicin; (5) Spermidine conjugate; (6) Sinapic acid; (7) Sinapoyl malate; (8) Disinapoyl-dihexoside; (9) Kaempferol-disinapoyl-trihexoside; (10) Disinapoyl-dihexoside (isomer of 8); (11) Disinapoyl-hexoside; (12) Trisinapoyl-dihexoside; (13) Trisinapoyl-dihexoside (isomer of 12); (14) Methyl sinapate. **B** Chemical composition of broccoli extract pre-and post-treatment with S9 fraction. The percentage area under the peaks in the HPLC chromatograms was used to quantitatively compare the compositions of the pre-and post-treatment extracts shown as bar charts and with identities color-coded at right of the bar charts, with compound identity listed on the right side of each bar. The fifteenth bar at the bottom of the chart represents other cumulative small molecules

An HPLC–UV-MS/MS method was adapted for the analysis of the OE and the resulting chromatograms are shown in Fig. 3A. Retention time (Rt), UV absorption maxima, MS^1^ and MS^2^ data, both in positive and negative mode, were analyzed and compared with literature data [15, 56–61] for tentative identification of the metabolites (Tables 1 and 2). Many of the identified constituents are conjugates of different numbers of sinapoyl, sugar, and flavonoid moieties, eluting according to their polarity. Three flavonoid (kaempferol) derivatives bearing sinapoyl groups (peaks 1, 2, and 9) displayed a characteristic UV absorption maximum at 268 nm. Two nitrogen-containing ingredients, neoglucobrassicin (peak 4, a glucosinolate) and spermidine conjugate (peak 5, a polyamine), were also observed. Remaining structures were identified as sinapic acid derivatives. In the MS^1^ spectra of these compounds, deprotonated molecules [M−H]^−^ were commonly observed in negative ion mode, whereas several positively charged species revealed a loss of 224 amu, indicating the loss of sinapic acid moiety. Similar MS^2^ fragmentation patterns were observed for these compounds, indicating structural relationship. Due to the limitation of MS interpretation, the exact identities of the sugars and the glycosidic linkages could not be determined.

**Table 1:** Peak identification by HPLC–UV-MS/MS analysis for the OE

Peak	Identification	Rt (min)	UV max (nm)	MS1 (−) m/z	MS2 (−) m/z	MS1 ( +) m/z	MS2 ( +) m/z
1	Kaempferol-sinapoyl-tetrahexoside	7.4	222, 268, 331	1139 [M−H] 569 [M−2H]^2−^	[1139] 977, 771, 547, 223	1141 [M+H]^+^ 531	[1141] 531, 369, 351, 287, 207, 175
2	Kaempferol-sinapoyl-trihexoside	7.9	223, 268, 331	977 [M−H]^−^	[977] 815, 609, 591, 446, 284, 205	979 [M+H]^+^	[979] 449, 369, 351, 287, 207, 175, 127
3	Sinapoyl-hexoside	8.5	235, 331	385 [M−H]^−^	[385] 325, 265, 247, 223, 205, 190, 175	795 [2M+Na]^+^	[795] 409, 247, 207, 185
4	Neoglucobrassicin	11.7	223, 268, 299, 320^a^	477 [M−H]^−^	[477] 446, 259	–	–
5	Spermidine conjugate^b^	13.0	225, 295	494 [M−H]^−^	[494] 479, 462	496 [M+H]^+^	[496] 479, 478, 425, 408, 351, 325, 283, 268, 232, 216, 191, 175, 149
7	Sinapoyl malate	15.0	236, 329	339 [M−H]^−^ 679 [2 M−H]^−^	[339] 223 [679] 339, 223	703 [2M+Na]^+^	[703] 363, 229, 207, 175, 157
8	Disinapoyl-dihexoside	20.0	226, 329	753 [M−H]^−^	[753] 529, 265, 247, 223, 205, 190	531 [M−224+H]^+^ 777 [M+Na]^+^	[531] 369, 351, 225, 207, 175, 147, 127
9	Kaempferol-disinapoyl-trihexoside	20.5	225, 268, 329	1183 [M−H] 591 [M−2H]^2−^	[591] 609, 652, 488, 407, 367, 325, 284, 265, 205, 223	1185 [M+H]^+^ 1207 [M+Na]^+^	[1185] 655, 369, 351, 287, 207, 175
11	Disinapoyl-hexoside	23.4	226, 329	591 [M−H]^−^	[591] 367, 352, 223, 205	369 [M−224+H]^+^	[369] 207, 175
12	Trisinapoyl-dihexoside	24.3	226, 326	959 [M−H]^−^	[959] 735, 529, 511, 247, 223, 205	737 [M−224+H]^+^	[737] 369, 351, 207, 175, 127

^a^UV profile could be affected by a coeluting minor component

^b^ Structure reported by Baumert et al. [56]

**Table 2:** Peak identification by HPLC–UV-MS/MS analysis for the S9-treated OE extract

Peak	Identification	Rt (min)	UV max (nm)	MS1 (−) m/z	MS2 (−) m/z	MS1 (+) m/z	MS2 (+) m/z
1	Kaempferol-sinapoyl-tetrahexoside	7.4	222, 268, 331	1139 [M−H] 569 [M-2H]^2−^	[1139] 977, 771, 547, 223	1141 [M+H]^+^ 531	[1141] 531, 369, 351, 287, 207, 175
2	Kaempferol-sinapoyl-trihexoside	7.9	223, 268, 331	977 [M−H]^−^	[977] 815, 609, 591, 446, 284, 205	979 [M+H]^+^	[979] 449, 369, 351, 287, 207, 175, 127
3	Sinapoyl-hexoside	8.5	235, 331	385 [M−H]^−^	[385] 325, 265, 247, 223, 205, 190, 175	795 [2M+Na]^+^	[795] 409, 247, 207, 185
4	Neoglucobrassicin	11.7	223, 268, 299, 320^a^	477 [M−H]^−^	[477] 446, 259	–	–
6	Sinapic acid^b^	13.7	227, 322	–	–	225 [M+H]^+^ 247[M+Na]^+^ 207	–
7	Sinapoyl malate	15.0	236, 329	339 [M−H]^−^ 679 [2M−H]^−^	[339] 223 [679] 339, 223	703 [2M+Na]^+^	[703] 363, 229, 207, 175, 157
8	Disinapoyl-dihexoside	20.0	226, 329	753 [M−H]^−^	[753] 529, 265, 247, 223, 205, 190	531 [M−224+H]^+^ 777 [M + Na]^+^	[531] 369, 351, 225, 207, 175, 147, 127
9	Kaempferol-disinapoyl-trihexoside	20.5	225, 268, 329	1183 [M−H] 591 [M−2H]^2−^	[591] 609, 652, 488, 407, 367, 325, 284, 265, 205, 223	1185 [M+H]^+^ 1207 [M+Na]^+^	[1185] 655, 369, 351, 287, 207, 175
10	Disinapoyl-dihexoside (isomer of 8)	22.0	225, 333	753 [M−H]^−^	[753] 529, 265, 247, 223, 205, 190	777 [M+Na]^+^ 531 [M−224+H]^+^	[777] 553, 467, 329, 301, 245, 207, 175
11	Disinapoyl-hexoside	23.4	226, 329	591 [M−H]^−^	[591] 367, 352, 223, 205	369 [M−224+H]^+^	[369] 207, 175
12	Trisinapoyl-dihexoside	24.3	226, 326	959 [M−H]^−^	[959] 735, 529, 511, 247, 223, 205	737 [M−224+H]^+^	[737] 369, 351, 207, 175, 127
13	Trisinapoyl-dihexoside (isomer of 12)	27.4	225, 326	959 [M−H]^−^	[959] 735, 511, 223, 205	737 [M−224+H]^+^	[737] 369, 351, 225, 207, 175, 147,127
14	Methyl sinapate	28.8	226, 324	–	–	239 [M+H]^+^ 261 [M+Na]^+^ 207	[239] 207, 192, 175, 147, 119, 91

^a^UV profile could be affected by a coeluting minor component

^b^Or derivative

The S9-treated OE resulted in both quantitative and qualitative modifications of the metabolites. The post-treated extract displayed several differences in composition and proportions of the components, compared to the untreated OE (Fig. 3A). For example, Peaks 6 (sinapic acid), 10 (a disinapoyl-dihexoside), 13 (a trisinapoyl-dihexoside) and 14 (methyl sinapate) appeared after S9 microsome fraction digestion, whereas the intensity of peak 7 (sinapoyl malate) was decreased. The relative intensities of flavonoid derivatives, especially peaks 1 and 2, increased in the S9-treated OE sample. Figure 3B presents a comparison of relative quantities of the identified constituents before and after S9 digestion. Clearly, sinapoyl malate (peak 7) was depleted during metabolism, whereas the kaempferol sinapoyl glycosides (peaks 1 and 2) increased.

## Discussion

*Brassica oleracea* var. italica (Broccoli) came to prominence in plant chemical utility by the Talalay lab, in the case of the production of isothiocyanates for cancer prevention [52, 62, 63]. Over the last 20 years, many plant types of the Brassicaceae have been indicated as part of an anti-inflammatory diet [64]. Sulforaphane is a broccoli metabolite known and implicated in anticancer activity [65–68]. However, the effects observed herein are not due to sulforaphane (not found in the OE or S9-treated OE) where the extraction method favored phenylpropanoids (sulforaphane not extracted or detectable in our method). Broccoli in its typical form for food consumption is not sufficient to target pain as desired chemicals are not induced or are too low in concentration. Broccoli is in the same family as *Arabidopsis thaliana*, a plant from which we have identified critical chemicals that could act as pain-targeting agents [15]. Broccoli produces phenylpropanoids relevant to inflammation [29, 69–74], which have been enhanced in the growth regime described herein. Extracts or simple chemical combinatorials can be more chemically stable and more effective than specific single chemical isolates [26, 28, 30]

HPLC-UV-MS/MS methods are commonly used for detection and tentative identification of components in complex plant extracts. UV detection with a diode array detector coupled with electrospray ionization mass spectrometry (DAD-ESI-MS^n^) in negative mode is a powerful tool for the analysis of phenolic compounds in botanical extracts, including extracts from Brassicaceae species [58]. In this study, the analysis parameters, such as the mobile phase gradient, UV detection wavelength and mass range, were optimized for efficient separation and detection of the phenylpropanoid constituents of broccoli extract. Several herein identified sinapic acid derivatives have been detected in other Brassicaceae plants, such as *Brassica oleracea* var. *costata* [59], *Brassica napus* [56, 60, 61] *Brassica juncea* [58] and *Arabidopsis thaliana* [15]. Sinapic acid and its esters are characteristic for the members of the Brassicaceae family. Sinapic acid is produced in the shikimate/phenylpropanoid pathway and then enzymatically converted into *O*-ester conjugates [58]. Sinapoyl-glucose (hexose) and sinapoyl malate are components of a well-known pathway of sinapate esters. Gentiobiose (dihexose) esterified with sinapate, as well as di- and trisinapate esters, are known to accumulate in broccoli [56]. Flavonoids, such as quercetin and kaempferol, can also be a part of sinapoyl conjugates. Kaempferol-sinapoyl-glycosides are widely distributed in Brassicaceae species [58], and they were detected in the studied broccoli extract. Apart from the sinapic acid derivatives, other constituents of the broccoli extract were reported herein. The spermidine conjugate has been identified in *Brassica napus* [56]. While sulforaphane, an isothiocyanate breakdown product of a glucosinolate glucoraphanin [75], was not detected in this study, another glucosinolate—neoglucobrassicin [57]—was found to be present. Treatment of the OE using liver S9 microsome fraction resulted in the modifications of metabolite composition. The relative amount of flavonoid sinapoyl glycosides increased after S9 treatment. As flavonoids are known for their anti-inflammatory properties [76], this change could support the observed effects of the S9-treated OE in reducing pain behavior in the mouse pain model, compared to those observed for the OE, even though OE was still effective.

The increase in opioid and narcotic deaths [7] despite the reduction in prescriptions [6], and the fact that pain is associated with suicide [77] indicates urgency is needed to accelerate the development of alternative therapies that can alleviate pain. Pain is often associated with inflammation and inflammatory disease, where current treatments do not guarantee effective pain relief. For example, cancer is known to cause persistent pain during and after treatment [78]. Many cancer survivors (approximately 50%) live with chronic pain post-treatment [79].

Ibuprofen, naproxen, and celecoxib are among the most consumed over-the-counter NSAIDs. Although effective for mild pain, long-term consumption is known to result in adverse effects including multi-organ toxicity [80]. Additionally, the increased dosage of NSAIDs may result in no therapeutic gain due to having a ceiling effect [80]. Over-the-counter analgesics such as acetaminophen result in hepatotoxicity with higher doses [81]. Increased dosage and/or long-term consumption of acetaminophen leads to many adverse effects that could exacerbate currently experienced pain [82]. Certain groups are especially at risk, such as pregnant women and hypertensive patients, where there is an increased risk of developmental disorders [83], and cardiovascular disease [84], respectively. Evidence of efficacy of acetaminophen regarding management of cancer pain is debatable [85]. Thus, it is important to invest in alternative solutions that do not result in increased complications and morbidity of pain-associated conditions.

Plant-derived phenylpropanoids can be potent interventions in inflammatory-based diseases and cancer therapy [76, 86–88]. Among the commonly tested phenylpropanoids, flavonoids are shown to target inflammatory pathways, and cancer metabolism, and induce cancer cell apoptosis [76, 86–88]. Polyphenols are shown to have a synergistic effect when combined with chemotherapeutics [76]. The mechanism of targeting inflammation is suggested to be through modulating NF-kB and Nrf2 signaling and ER-related stress pathways [86]. A commonly tested flavonoid, quercetin, induces its anticancer activity by modulating VEGF, apoptosis, PI3K/Akt/mTOR, and Wnt/β- catenin signaling pathways, in addition to inhibiting the inflammatory enzymes of the cyclooxygenase (COX) pathway and other inflammatory mediators, thus reducing the risk of cancer [89, 90]. A phenylpropanoid extract derived from Arabidopsis mutants reduced inflammatory marker levels associated with APOE4 allele-induced neuroinflammation, where APOE4 is a major risk for Alzheimer’s disease [15]. Developing a strategy to produce an suitable extract from edible plants might present additional benefits such as increased bioavailability. Studies have reported variations in bioavailability among polyphenols [29]. This is especially prevalent for single-isolate compounds, where resveratrol’s bioavailability and solubility hindered its potency [26]. Factors such as processing and metabolism are also reported to result in variable polyphenol bioavailability and thus its potency [25]. Our method herein indicates while effects are different for liver-processing (using S9 microsome fraction), both OE and S9-treated OE have anti-inflammatory results, both in vitro and in vivo, indicating bioavailability.

This project was to find a broccoli type that could be a candidate to develop natural anti-inflammatories, with comparable phenylpropanoid groups similar to that reported for Ghura et al. [15]. A strength of the study included that we tested many conditions before we arrived at the featured protocol in this manuscript, where broccoli seedlings can present as anti-inflammatory powerhouses in a way previously unexplored. While the world is familiar with sulforaphane, very little is studied regarding sinapates and flavonoids action in states of inflammation. Another strength was that data indicated that in addition to anti-inflammatory activity, OE-treated injured discs showed higher expression of specific collagens, indicating the potential for disc regeneration. A direction to pursue in addition to further characterizing anti-inflammatory effects is to understand this duality of opposing inflammation, and promoting repair.

A shortcoming of the present study that we could not utilize a number of cultivars that we would have liked to explore, due to the common broccoli issue that it is typically an out-crosser (self-incompatible). Hence, it was a challenge to find modern cultivars that could be reliable sources from batch to batch, or population to population. However, Calabrese being a non-GM heirloom, affordable seed, and having good reproducibility in terms of growth, is promising. Abiotic induction and extraction of potential medicinal chemicals was reliable. Another shortcoming is that pain and inflammation manifest in a number of ways, and it was not possible to test many types of pain. We focused on specific common conditions as an initial study. Hence the heirloom variety Calabrese shows promise for further lab and clinical studies on inflammation and tissue repair, with the goal of developing an affordable anti-inflammatory therapeutic.

## Conclusion

Sinapates and flavonoids of the phenylpropanoids have activities that indicate promising therapeutic directions. Herein, we presented a potential solution to several problems caused by the currently available medications for pain and inflammation—a plant-based method that is low cost, and non-addictive. Such interventions can be efficient and cost-effective for many inflammatory-based diseases. As a plant is a combinatorial of chemicals, future studies can also include doing anti-inflammatory assays with one or more chemicals found in the S9-treated OE or OE, in order to find out which chemicals, singly or in combination, are required for effects observed herein.

### Supplementary Information

**Additional file 1: Table S1.** Initial heirloom broccoli screen of estimated phenylpropanoids^1^ in fresh weight seedling tissue^#^. **Figure S1.** Process of method to develop extracts.
