# Supplementary material for: Phenylpropanoid-enriched broccoli seedling extract can reduce inflammatory markers and pain behavior
Source: J Transl Med. 2023 Dec 19;21:922. doi: 10.1186/s12967-023-04777-1 (PMC10731810; doi:10.1186/s12967-023-04777-1)
Supplement: Supplementary file 1 — Additional file 1: Table S1. Initial heirloom broccoli screen of estimated phenylpropanoids1 in fresh weight seedling tissue#. Figure S1. Process of method to develop extracts. [file 12967_2023_4777_MOESM1_ESM.docx]

**Table S1. Initial heirloom broccoli screen of estimated phenylpropanoids^1^ in fresh weight seedling tissue^#^.**

| Heirloom Broccoli type | Sinapic esters* | Polyphenols |
| --- | --- | --- |
| Calabrese | .32 | .21 |
| De Cicco | .24 | .11 |
| Purple Sprouting | .22 | .20 |
| Rudolph | .19 | .14 |
| Waltham | .18 | .13 |
| Romanesco | .18 | .13 |

^1.^ Estimated phenylpropanoid peak area is proportional to metabolite quantity and is just a first screen after UV (317 nm, 254 nm) and cold induction (1 h at 4^o^C), estimated on BioRad SmartSpec^TM^ Spectrophotometer, confirmed by fluorescence

Chlorophylls were negligible

^#^extraction from fresh seedling leaves

*Peak association with metabolites based on wavelength and ethanol extraction [38]. Extraction solubles based on plant materials and conditions described in Methods.

**S1**

**Fig. S1. Process of method to develop extracts.** Seeds of heirloom cultivars of *Brassica oleracea* var. italica (Broccoli) shown in Table S1 were grown on 0.5X MS minimal medium agarose. At age 5 d, samples were treated with abiotic stimuli (see Methods). Seedling leaf tissue was harvested 12 h after the last abiotic treatment into 90% ethanol for fresh weigh extraction (Table S1), or after Calabrese was identified as most useful for phenylpropanoid production, liquid nitrogen and then ground to a fine powder. Extracted (see Methods) tissue was dried, and when ready for testing, resuspended in DMSO until dissolved as described in Methods. Chemical species were determined by absorption spectra and analytical chemistry of the original extract (see OE; Methods). OE was further processed by treatment with S9 fraction (Methods) for in vivo mouse pain behavior study.

**A. Pain behavior (licking injected paw) comparison of male and female responses: treatment with OE.** Mice were treated as described in Fig. 2 for OE, then data was analyzed by sex and assessed for the difference of mean in unpaired t-test as described in Methods. Male n=4, female n=4.

**S2**

**A**

**Late (40-55 min)**

**Early (0-5 min)**

**B. Pain behavior (licking injected paw) comparison of male and female responses: treatment with S9.** Mice were treated as described in Fig. 2 for S9, then data was analyzed by sex and assessed for the difference of mean in unpaired t-test as described in Methods. Male n=4, female n=4.

**B**

**Late (40-55 min)**

**Early (0-5 min)**
